# Acute Tonsilitis

**Published:** 1890-04-12

**Authors:** 


### ACUTE TONSILITIS.

Some remarks by Dr. Andrew Dunlop, physician to the Jersey Dispensary, on "Acute Follicular Tonsilitis," are well worth notice, as tending to establish a better diagnosis between diphtheria and tonsilitis. Dr. Dunlop observes that in typical cases the disease commences with malaise and rigors and sore throat. The tonsils are found reddened and swollen, and two or three rounded yellowish-white spots may be seen. There may also be a swollen gland under the angle of the jaw. On the third day the symptoms are generally on the decline, but in some instances the secretion spreads itself over the tonsils, with increase of other symptoms. In many cases there is stiffness of the nose. When a case of follicular tonsilitis is seen, where one or both tonsils is covered or partly covered with secretion, there is striking resemblance to diphtheria. It is found, however, that the friable material is different from diphtheritic membrane, and may be easily removed. Again, like diphtheria, this malady often appears in an epidemic form. It is, therefore, highly probable that it is infectious. Although Dr. Dunlop does not regard this malady as diphtheria, he remarks that the two seem closely allied. "It appears probable that it is nearly related to diphtheria, much as if it were another species of the same genus." Now we have several times remarked (*vide* "Retrospect," December 14th), when noticing reported success by various modes of treatment of diphtheria, that physicians are not at all agreed as to what constitutes diphtheria. No doubt those who have reported very successful treatment by the use of certain remedies, have included cases which Dr. Dunlop describes under the term "Acute follicular tonsilitis." Diphtheria is a term of comparatively recent origin for maladies which formerly were called by more homely names. The questions are whether we should include all cases of sore throat with accompanying exudation or deposit under the term diphtheria, or whether acute follicular tonsilitis, which would cover much ground, should, as advocated by Dr. Dunlop, "be accorded its proper place in our nosology?" Perhaps some authority or other will decide this query. Then we should know what confidence to place in the reported recurring successful treatment of diphtheria by various agents.

